# Utilising genomic association data for causal inference in anorexia nervosa

**DOI:** 10.1007/s00335-025-10150-y

**Published:** 2025-07-10

**Authors:** Danielle M. Adams, Murray J. Cairns

**Affiliations:** 1 Centre for Complex Disease and Precision Medicine, School of Biomedical Sciences and Pharmacy, The University of Newcastle, Callaghan, NSW Australia; 2 Precision Medicine Research Program, Hunter Medical Research Institute, Newcastle, NSW Australia

**Keywords:** Anorexia nervosa, Genome wide association study, Mendelian randomisation, Causal inference, Gene association, Genetic correlation

## Abstract

Anorexia nervosa (AN) is a prevalent psychiatric disorder with high rates of mortality and limited treatment options. AN is a complex disorder, for which common variation contributes to disorder risk. To dissect the genetic architecture of AN, a variety of statistical methods can be applied. Many of these utilise genome-wide association study (GWAS) datasets to investigate biological mechanisms within disease progression in addition to broader associations between complex traits. GWAS for AN have revealed important biological insights, however, these have not translated into new pharmacotherapies. Here, we review the application of statistical methods that use GWAS, to investigate the relationship between genetic variation, biochemical compounds and complex traits to identify potential relationships which could advance our understanding of disease biology. We discuss genetic variant association data for AN, the application of gene-based and complex trait level correlation methods and approaches for establishing evidence of causality between complex traits and AN. These methods all contribute to the growing literature regarding the genetic influences of AN risk and demonstrate that statistical analysis utilising genetic data is a valuable tool to progress our understanding of this disease.

## Abstract (plain language summary)

Anorexia nervosa is a psychiatric disorder influenced by both genetic and environmental components. Aspects of anorexia nervosa include excessive energy restriction, fear of gaining weight and an inaccurate perception of oneself. Consequences of this disorder can be fatal, and no current medications are approved to target the underlying causes of the disease. The recent availability of data dissecting genetic influences on the disorder provides a promising avenue to identify potential treatment opportunities which are desperately needed. This data, which contains information about millions of genetic variants and how they relate to disease risk, can be leveraged to analyse potential risk factors and biological pathways related to the disease. However, these analyses have yet to eventuate into clinically validated treatments. Here we present a summary of recent progress in the use of genetic data to identify potential treatment targets for anorexia nervosa. Additionally, we present which risk factors have the most evidence to support their potentially causal role in anorexia nervosa risk.

## Introduction

### Background

Anorexia nervosa (AN) is a complex and polygenic disorder which means it is influenced by various genetic and environmental components (Watson et al. 2019). Complex disorders including AN, can have devastating consequences for both individuals and communities due to their negative health complications, financial costs and often chronic symptoms (Johansson et al. 2023). Health outcomes in AN are poor and available treatments do not meet the needs of patients (Crow 2019; Edakubo and Fushimi 2020; Watson et al. 2019). One study found that at a 6-year follow-up, 26% of AN patients in remission relapsed into an eating disorder (Castellini et al. 2011). Despite immense research, the diversity of risk factors, their degree of influence and the relationship between them remains unknown for many traits associated with AN. Confounding present within observational studies and the expense required to undertake clinical trials can inhibit progress in this field in some cases. The utility of genome information presents an opportunity to investigate this disorder through a variety of methods with different benefits and limitations (Park et al. 2022).

### Anorexia nervosa

AN is a disorder involving disturbed eating habits, anxiety around food and an altered self-perception (2013). Within the Diagnostic and Statistical Manual of Mental Disorders (DSM) version 5, there are three diagnostic criteria used to define AN (2013). (1) That an individual restricts their energy intake such that their body mass index (BMI) is below expectation relative to other phenotypes such as age and sex. (2) Either possessing an intense fear of gaining weight or interfering with weight gain despite a low weight. (3) Inaccurate recognition of body shape or excessive connection between body weight or shape and self-evaluation. Two subtypes are defined for this disorder relative to the mechanism through which the individual restricts/reduces their weight. The restriction subtype involves reduced food intake and excessive exercise whereas the binge-eating/purging subtype involves (within the last three months), inducing vomiting or ingesting laxatives, but may otherwise appear to eat appropriately. Unless otherwise indicated, studies that mention AN typically refer to the restriction subtype. The severity of the disorder is classified based on BMI, where the most severe level is BMI < 15. The prevalence of AN and other eating disorders has been increasing over the last decade, with the Global Burden of Disease Study citing a 6.1% increase in ‘years lost to disability’ for AN from 2007 to 2017 globally (2018). For AN, occurrence is increasing amongst individuals under 15 years old, while it is currently estimated that 4% of females and 0.3% of males will be diagnosed during their lifetime (van Eeden et al. 2021). The disorder primarily begins in younger individuals with typical onset before the age of 18 (Watson et al. 2022). Finally, the relatively smaller proportion of males diagnosed with AN inhibits any progress toward a male-stratified analysis. Mortality rates for AN are substantial, estimated between 2.8 and 5% - typically resulting from malnourishment or suicide, highlighting the pressing need for action (Edakubo and Fushimi 2020; Fichter et al. 2017). Consequences of AN can be varied and extend beyond psychiatric symptoms and reduced energy availability. These include an increased risk of bone disease due to reduced bone mineral density, gastrointestinal complications with differing microbiota compositions, amenorrhea, bradycardia and bronchiectasis, some of which are side effects of malnourishment (Puckett et al. 2021). Treatment is often inhibited by psychiatric co-morbidities and a desire within some AN patients to avoid actions that might increase their weight (Abbate-Daga et al. 2013) and even when individuals are actively seeking treatment few options are available. The current treatment practice for AN is to address the psychological aspects of the disease through cognitive behavioural therapy (Linardon 2018), some drugs such as fluoxetine may be prescribed off-label (Rodan et al. 2023) although no medications are approved to address the underlying biology of the disorder (Reas and Grilo 2021). For severe cases of AN which may involve hospitalisation, refeeding strategies may be implemented, which aim to increase weight by approximately 2 pounds per week (Mehler et al. 2010). A randomised control trial (RCT) in 2021 investigated outcomes in AN patients who underwent a high vs. low-calorie refeeding strategy (Golden et al. 2021). Over one year they observed no significant difference between the two strategies, however, both strategies exhibited high rates of rehospitalisation (32–35%). Furthermore, a lethal potential side effect of refeeding severely malnourished individuals is ‘refeeding syndrome’ which can induce stress on the cardiovascular system (Mehanna et al. 2008). Thus, novel risk factors need to be identified which may lead to improved treatment outcomes.

### Genome-wide association studies

Genome-wide association studies (GWAS) are used to estimate the relationship between genetic variation and a trait of interest, including measured quantitative traits and binary traits (e.g., disease liability) (Haines et al. 2005; Loos 2020). This hypothesis-free method tests the association between millions of variants across the genome and a trait of interest. Typically, this method is applied to common genetic variation, such as common single nucleotide polymorphisms (SNPs) and insertions/deletions, due to the increased power to detect common variant associations in most instances (Auer and Lettre 2015). However, this genome-wide approach can also apply to rare variation and structural variation (Uffelmann et al. 2021). Herein, common frequency variation is used to refer to loci that occur in the population with at least 1% frequency (Goswami et al. 2021). Typically, common variants exhibit smaller effect sizes compared to rare variation, particularly exonic rare variants. Whilst their individual effect sizes are often low, the combined effect of many common variants contribute a larger, polygenic component of complex trait genetic risk. Specifically, complex traits are polygenic, meaning they are influenced by many genetic factors as opposed to traits caused by variation in a single gene (Lvovs et al. 2012). GWAS has revealed the polygenic nature of many complex traits by identifying multiple significantly associated variants. While this data has provided insights into many diseases, the challenge remains to convert these findings into biologically salient results which could lead to improved patient outcomes.

### Genome-wide association studies performed on anorexia nervosa

GWAS has previously been used to identify genetic signals associated with AN. AN is comprised of both environmental and genetic components, with heritability estimates ranging from 48 to 74% (Schaumberg et al. 2017). Heritability refers to the proportion of phenotypic variation explained by genetic information (Hagenbeek et al. 2023). The upper bound of potential heritability is often estimated through a twin study which compares the frequency or level of a phenotype between monozygotic and dizygotic twins to determine the degree of genetic influence on this trait. A component of this heritable risk referred to as SNP heritability, is the variance which can be explained in the phenotype due to the influence of common SNPs identified from GWAS. While high heritability is not a prerequisite of GWAS, this does provide the theoretical upper bound of the SNP heritability these GWAS can estimate (Huang et al. 2024). Initial GWAS for AN had low sample sizes which did not identify any variants significantly associated with the disease, however, the last two GWAS with larger numbers of cases identified one (Duncan et al. 2017) and eight (Watson et al. 2019) independent risk loci for AN. Six AN GWAS have been performed at the time of writing, estimating disease association with common variation (Table 1). In addition, another GWAS estimating disease association with microsatellite markers was performed in 2009 (Nakabayashi et al. 2009). One study performed a GWAS on the age of onset of AN, and while this did not reveal any genome-wide significant variants, it suggested a potential association with the age of menarche (Watson et al. 2022). This study also performed a GWAS on typical age of onset AN and identified two independent risk loci, one of which was unique to this study (rs4641158). The FinnGen consortium has performed GWAS on several eating disorders, including AN and bulimia nervosa, although these have been underpowered with fewer cases than the 2014 AN GWAS (Kurki et al. 2023). These analyses have revealed variants associated with AN which are estimated to explain up to 20% of phenotypic variability. Further analysis is required to investigate the associations between single variants, disease and their biological implications.

For some complex traits, analysis methods that further leverage the results of GWAS have made important contributions to understanding the biological processes of these diseases to progress current treatment practices (Visscher et al. 2017). Genetic data supporting the development of drugs increases the probability that the investigated drugs will be successful in their application (Minikel et al. 2024). GWAS for Crohn’s disease and ulcerative colitis have identified genetic variants related to interleukin-23 as risk-increasing (Sewell and Kaser 2022). This contributed to the investigation of drugs that inhibit this interleukin in the treatment of these disorders (Parigi et al. 2022). Additionally, GWAS for auto-immune disorders revealed associations with major histocompatibility complex genes; *ERAP1* and *ERAP2* (Agrawal and Brown 2014), after which drug development began to target these genes (Camberlein et al. 2022). Despite the availability of genetic association data for AN, the therapeutic utility of this data has yet to be utilised in a substantial way. This chapter discusses the progress made towards understanding the genetic and biological mechanisms of AN through leveraging GWAS summary statistics. The methods used to understand the effect of individual variants, genes and pathways on disease as well as the relationship between associated traits through genome-wide approaches are outlined. Additionally, the strengths and limitations of these methods and future work that should be pursued to enhance the clinical relevance of these studies for AN are described.

**Table 1:** Genome-wide association studies (GWAS) for anorexia nervosa

GWAS publication year	2010 (Wang et al. 2011)	2014 (Boraska et al. 2014)	2017 (Duncan et al. 2017)	2019 (Watson et al. 2019)	2021 (Watson et al. 2022)	2023 (Kurki et al. 2023)
Number of cases	1033	2,907	3,495	16,992	6,998	452
Number of controls	3,733	14,860	10,982	55,525	25,042	411,729
Number of SNPs investigated	610,000	1,185,559	10,641,224	9,020,081	--	16,962,023
Number of significant independent SNPs	0	0	1	8	2	0
Most significant SNP: associated gene(s)	rs6959888: ZNF804B	rs9839776: SOX2OT	rs4622308: IKZF4, RPS26, ERBB3, PA2G4, RPL41, and ZC3H10	rs9521797: *NCKIPSD*	rs3821875; CELSR3	rs762643080; LINC01179
Study populations	European	European and Japanese ancestry	European ancestry	European ancestry	European ancestry	Finnish ancestry
SNP heritability	--	--	20%	11–17%	17–25%	--
Power	--	80% for 1.15 OR	83.1% for 1.25 OR	80% to detect 0.9% lifetime risk	80% power to detect SNP-h2s ≥ 0.03	--
Study groups	CHOP	GCAN & WTCCC3	CHOP/PFCG & GCAN/WTCCC3 & PGC	ANGI & PGC-ED & GCAN & WTCCC-3 & UKBB	ANGI & WTCCC3/GCAN & CHOP/PFCG	FinnGen
Test statistic inflation (λ)	1.08	1.03	1.080	1.22	--	0.99

The six GWAS for anorexia nervosa (publication year: 2010, 2014, 2017, 2019, 2021, 2023) which measure the association between common single nucleotide polymorphisms (SNPs) and anorexia nervosa status. Rows indicate the characteristics and results of each GWAS. Study groups; Children’s Hospital of Philadelphia (CHOP), the Price Foundation Collaborative Group (PFCG), Genetic Consortium for Anorexia Nervosa (GCAN), Wellcome Trust Case-Control Consortium-3 (WTCCC-3), Psychiatric Genetics Consortium (PGC), PGC-Eating disorders working group (PGC-ED), United Kingdom Biobank (UKBB), Anorexia Nervosa Genetics Initiative (ANGI). Power was reported by the respective GWAS publication.

## Main text

### Genome-wide associated signals for anorexia nervosa

**Fig. 1:**
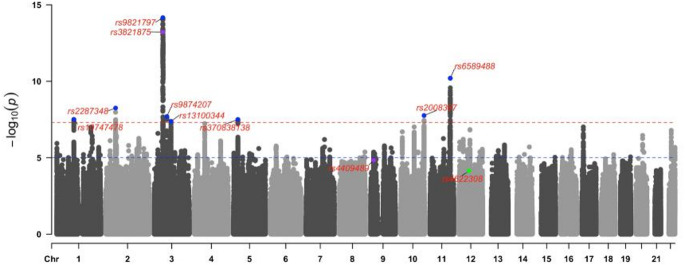
Manhattan plot of anorexia nervosa SNP associations from the 2019 GWAS. Points represent individual SNPs; highlighted and labelled points indicate SNPs that reached genome-wide significance from either the 2021 (purple), 2019 (blue) or 2017 (green) GWAS for anorexia nervosa (Table 2). The SNP; rs4641158, was not reported in the 2019 GWAS, thus a nearby SNP; rs4409489 which shares the same closest gene (based on transcription start site) is mapped here. Chromosomal position is indicated on the x-axis and -log base 10 p value (-log 10 (P)) significance is indicated on the y-axis. The red horizontal line indicates genome-wide significance at *P* = 5 × 10^-8^ while the blue line indicates suggestive significance at *P* = 1 × 10^-5^. Manhattan plot created with anorexia nervosa GWAS summary statistics (Watson et al. 2019) using the CMplot package in R (Yin et al. 2021)

One piece of information provided by a GWAS, which is used frequently within statistical genetics methods, is the association between genetic variants and the trait of interest. These loci are designated genome-wide significant when their p value is less than that of the conventional genome-wide multiple testing threshold (*P* < 5 × 10^− 8^) (Dudbridge and Gusnanto 2008). Genome-wide associations are valuable since they are statistically more likely to reflect a true association with the trait studied; however, careful consideration should be given to the interpretation of these loci. Nearby variants are often inherited together at an increased rate [linkage disequilibrium (LD)], which gives rise to spurious associations between variants and traits due to correlation with causal variants in LD. Statistical approaches can be applied to distinguish the most likely causal variants from other variants in LD. Within the context of statistical genetics, variants may be described as independent. This refers to a particular variant in an analysis, where other variants in LD have been removed, and may be useful to indicate that two variants are not identifying the same signal. This may differ from the lead variant which is determined by the variant with the smallest p value. To date, variants from ten loci are significantly associated with AN through GWAS, where the lead variant is reported (Table 2, Fig. 1). The 2021 AN GWAS identified two novel lead SNPs of which one of these (rs3821875), was within a previously identified risk locus from the 2019 GWAS. The variant identified in the 2017 GWAS (rs4622308) was not replicated in the larger 2019 GWAS (Fig. 1), potentially due to study/population effect heterogeneity or Winner’s curse. Winner’s curse refers to a concept whereby when data (such as SNPs) are selected based on extreme values (for instance large test statistics), the true values for these data points may be less than what is reported (Xiao and Boehnke 2009). The degree of this bias will be dependent on the variability of the value measured and the sample size of the study.

The biological mechanisms of variants are not always well understood, however, they may be investigated by assigning variants to genes (Fridley and Biernacka 2011). From the eleven-genome-wide significant variants associated with AN, the first logical step is to identify the nearest gene through which these variants may exert an effect. However, the lead variant used for this mapping may not be the primary genetic factor responsible for the association due to LD. Finemapping is a Bayesian statistical approach which can be used to prioritise likely causal variants within a correlated set (Broekema et al. 2020). Bayesian statistics in comparison to frequentist statistics is differentiated by assigning a prior probability to a hypothesis of interest, which is updated to a posterior probability by integrating available data. Conversely, frequentist statistics assigns a probability to the data which is then compared relative to a null hypothesis to determine if there is sufficient evidence to reject (Hackenberger 2019). Multiple finemapping approaches exist, including those that prioritise variants based on their correlation (R^2^) with the lead variant, penalised regression of p values or Bayesian selection to update the posterior probability of a causal effect model (Schaid et al. 2018). Bayesian selection methods will assign a posterior probability that each variant has a causal influence on the trait of interest. Variants may then be grouped into credible sets which define the smallest group of variants required to capture the causal variant with a particular probability (Schaid et al. 2018). The prioritised variants from this approach may not necessarily match the lead SNP in the locus due to the patterns of LD in the region. Methods such as finemapping should be applied to AN so that subsequent biological interpretations are well-founded. Most GWAS signals exist outside of gene coding regions, thus another way to predict their functional mechanism is by identifying genes whose expression they may regulate (Hindorff et al. 2009). In some instances, the variant may exert a stronger effect on distant genes through mechanisms such as regulating the expression of mRNA (Table 2). Analysis to determine if variants are associated with changes in gene expression (eQTL) can enhance our evidence of which gene is functionally influenced as a direct consequence of that variant (Nica and Dermitzakis 2013). However, in some instances, multiple eQTL associations prevent the identification of a singular causal gene. For instance, the most significant AN GWAS variant (rs9821797) is localised to *NCKIPSD* based on transcription start site location while it is an eQTL for 19 other genes (Adams et al. 2023). Analyses of AN risk loci identified in the 2019 GWAS have been mapped to genes through location and expression data to enhance knowledge of disease risk. Specifically, these variants have been mapped to genes associated with varied phenotypes such as eosinophil percentage and whole-body fat mass (Watson et al. 2019).

**Table 2:** All genome-wide significant SNPs for anorexia nervosa

Genome-wide significant AN SNPs	Year	Nearest gene	Top three associated traits of AN SNP^+^
rs3821875	2021	*CELSR3*	*WDR6* expression *SLC26A6* expression Thioredoxin domain-containing protein 12 expression
rs4641158	2021	*CNTLN*	Posterior corona radiata No hearing problems Corneal resistance factor
rs9821797	2019	*NCKIPSD*	*WDR6* expression Thioredoxin domain-containing protein 12 expression *SLC26A6* expression
rs6589488	2019	*CADM1*	Heel bone mineral density Heel quantitative ultrasound index
rs2287348	2019	*ERLEC1*	*PSME4* expression *ACYP2* expression *CHAC2* expression
rs2008387	2019	*MGMT*	*MGMT* expression Standing height Creatinine level
rs9874207	2019	*FOXP1*	Corneal resistance factor Corneal hysteresis
rs10747478	2019	*PTBP2*	Arm fat mass Trunk fat mass Whole body fat mass
rs370838138	2019	*CDH10*	--
rs13100344	2019	*DHFR2*	--
rs4622308	2017	*ERBB3* ^*+*^	Height Eosinophil percentage Childhood onset asthma

Each independent genome-wide significant SNP for anorexia nervosa is indicated in the first column with the year it was identified. The third column shows the nearest gene based on transcription start site location in open targets genetics (Ghoussaini et al. 2021; Mountjoy et al. 2021) and the fourth column shows the top three IEU GWAS database (Elsworth et al. 2020) hits for which this SNP is also genome-wide significant. IEU GWAS was used for the rs4622308 nearest gene due to unavailable data in open targets genetics.

+ Data from IEU GWAS database

### Identifying associated genes

Methods which combine the effects of multiple variants into genes can increase the likelihood of signal discovery above that of individual GWAS. Gene-based approaches combine signals from several variants and thus can increase power for signal discovery where each signal would otherwise not reach significance thresholds within smaller sample size GWAS. Two primary methods that have been used to investigate the aggregate effect sizes from AN GWAS to genes are MAGMA (Multi-marker Analysis of GenoMic Annotation) (de Leeuw et al. 2015) and TWAS (Transcriptome-wide association study) (Gusev et al. 2016).

MAGMA estimates the association between genes and traits by assuming that variants act through the genes to which they are annotated, depending on their physical location. Thereby not assuming a specific biological mechanism of action of the variant upon the gene. MAGMA can provide evidence of which genes are most likely to be associated with disease, but it does not indicate the direction of association, only the statistical significance of the signal. At the time of writing, three studies have published MAGMA results for AN. The most recent AN GWAS performed MAGMA and identified 79 significant genes, varying in function and genomic location, however, most were within chromosome 3 and within genome-wide significant loci (Watson et al. 2019). Only one significant gene was identified from the other two MAGMA analyses, notably, both used the lower-powered, 2017 AN GWAS summary statistics rather than the 2019 GWAS. This gene, *KIT*, was identified in a gene association analysis of a meta-analysed AN and OCD GWAS (Yilmaz et al. 2020) and was not significant in the more recent analysis. The third paper only investigated dopaminergic and serotoninergic-related genes, rather than a broad selection across the genome and did not identify any genes significantly associated with AN (Cabana-Domínguez et al. 2022). When larger GWAS for AN become available, gene-based analysis could provide insight into dysregulated genes which may be involved in the mechanistic action of disease progression.

TWAS is an approach which leverages GWAS trait association data and imputed mRNA expression weights to estimate the association between predicted gene expression and trait risk. Independent cohorts are used with a variety of tissues available such as blood, brain regions and adipose, to estimate the association between genotype and mRNA expression. This data is used to train models of genetically regulated expression from which expression weights for variants are generated. These expression weights are integrated with the corresponding variant association data from the AN GWAS, to predict the association between genetically regulated gene expression and AN risk. This enables researchers to estimate the statistical significance and direction of association between genes with multiple traits from precalculated expression weights. Limitations of this approach include the lack of available gene expression weights for all genes and co-regulation of genes or LD between proximal genes (Reay and Cairns 2021). Moreover, an insufficient sample size of expression weight cohorts and the use of non-trait-specific tissues can result in biased estimates (Wainberg et al. 2019). This method can also be extended to the expression of other variables, such as protein expression and alternatively spliced mRNA. Few TWAS have been performed on AN at the time of writing. A TWAS was performed to investigate the spatial and temporal gene expression differences both within and between AN and OCD (Cheng et al. 2020), with the aim of investigating similarities between these highly associated disorders. Temporal gene expression was predicted using genetically regulated expression weights from six periods ranging from fetal development to adulthood. This identified 9 brain regions and 12 temporal stages which had signals significantly associated with AN. While this study did discuss the functional implications of dysregulation across brain regions, it did not consider specific mechanisms through which particular genes may influence AN risk. Two studies utilised PrediXcan, a specific TWAS method, differing in which mRNA tissues were utilised and each identified 36 (Watson et al. 2019) and 53 (Johnson et al. 2022) significantly associated genes. The primary difference between PrediXcan and other TWAS methods such as FUSION or MetaXcan is that the latter two use summary GWAS data rather than individual-level genotype data. Additionally, the prediction models differ between these approaches and FUSION which only utilises genes with statistically significant heritability estimates from *cis*-acting variants (termed *cis*-heritability) (Fryett et al. 2018). Cis-acting variants may operate through different regulatory mechanisms in comparison to trans-acting variants (Mattioli et al. 2020). The first of these studies did not elaborate on the results of their analyses, while the second of these studies performed a phenome-wide association study to identify associations between these genes and traits (Johnson et al. 2022). They identified the AN-associated genes which also displayed association with autoimmune diseases, cholesterol levels, tobacco use, pain scores and glucagon medication. They found evidence for BMI-gene expression interactions for three genes: *MGMT*, *CTNNB1* and *PROS1*, which are significantly associated with AN in other analyses (Adams et al. 2023; Watson et al. 2019). BMI level is intrinsically linked to AN since changes to BMI are a direct consequence of excessive food restriction and reduced BMI is an aspect of the diagnostic criteria for AN (2013). Thus, associations between genes and BMI should be investigated further to ensure that BMI is not a confounder in any associations identified.

Although these methods are associative and not necessarily causal, they can prioritise potentially causal risk factors by identifying significantly correlated genes, proteins, or mRNA. Further methods are required to investigate the presence of confounders within this relationship. Identifying causal genes for disease can be powerful since drugs exist which may directly interact with these genes or a pathway they function in. Due to the limited number of AN gene-based analyses and the diverging scopes of many of these analyses, most either did not identify significant signals or their most significant signals were not replicated across publications. Future analysis should focus on the biological implications of their results while relating these to potential treatment implications which could be explored in future analysis.

### Identifying associated pathways

To complement gene-based analyses and identify functional mechanisms of disease action, gene-set analysis can be performed. This involves grouping genes into sets of genes constituting a biological pathway with a known function. Within the self-contained gene-set analysis, test statistics from gene-based associations are combined to estimate the overall association of the pathway with the disease. Then within the competitive gene-set analysis, test statistics from the self-contained analysis are utilised to predict if genes within the set of interest are more strongly associated with the trait compared to other genes in the analysis (de Leeuw et al. 2015). MAGMAs gene-set analyses uses the MAGMA gene analyses results to estimate the association between a pathway and a trait of interest. For example, the most recent AN GWAS performed MAGMAs gene-set analysis and identified one significant pathway involving the regulation of embryonic development (Watson et al. 2019). The covariance of gene associations is used to adjust for LD between genes within a Generalized Least Squares model to estimate the pathways association with the trait. The limitations of MAGMA, such as indirect and unequal variant effects, still exist within the MAGMA gene-set analysis due to the use of the same methodological framework.

Similarly, a TWAS study generated its own pathway-prioritising TWAS method, JEPEGMIX2-P, using the 2017 AN GWAS summary statistics (Chatzinakos et al. 2020). They identified one pathway significantly associated with AN (response to double-stranded RNA), and while they compared this method to MAGMA they did not identify any pathways significant for MAGMA’s gene-set analysis. The pathway for the response to double-stranded RNA is involved in the response to viral infections which supports a role of the immune system in AN, replicated elsewhere (Adams et al. 2023; Gibson and Mehler 2019). However, more research is required to determine the specific aspect of the immune system involved in AN pathogenesis and its mechanism of action.

### Estimating the association between modifiable traits and anorexia nervosa using genome-wide association studies

In addition to targeting genes and pathways, higher-order complex traits can be investigated. Determining the causal relationship between complex traits can provide information about their underlying biological mechanisms leading to increased disease risk. While these traits may have more biological complexity to disentangle compared to the mechanistic effect of genes or pathways, some traits can have the advantage of being easily modifiable. For example, insulin levels can be increased directly through insulin injections or indirectly through diet changes. Genome-wide approaches, to investigate trait biology, can be applied to genetic data across traits to estimate associative or causal relationships and enhance evidence of a relationship between two traits. If there is a causal relationship between traits then correlation may follow, however, the inverse is not necessarily true, i.e. correlation between traits does not imply causality. Two traits may be genetically correlated but not causal due to a multitude of reasons including horizontal pleiotropy whereby a variant is associated with multiple factors through separate mechanisms and LD whereby two different variants in LD influence two separate traits, thus each variant appears to be associated with both traits (Kraft et al. 2020). Here, we discuss three methods that are used to estimate the association between genetic traits; linkage disequilibrium score regression (LDSC), latent causal variable (LCV) analysis and Mendelian randomisation (MR).

Methods such as LDSC (Bulik-Sullivan et al. 2015) utilise GWAS association data and a LD reference panel to measure the genetic correlation between two traits. LD scores for each variant are calculated by summing the extent of LD a variant demonstrates with other proximal variants (Ni et al. 2018). Additionally, genetic covariance, a measure of how two values change in relation to each other, is estimated from variant association data from both GWAS (Lu et al. 2017). Genetic correlation is then calculated by normalising the genetic covariance between two traits by the heritability estimates of each trait, both of which are calculated from LD and Z scores of variants throughout the genome (Pettit and Amos 2022). If two traits are correlated by this method, it can suggest that there is a shared biology between the two traits and potentially overlapping causal factors. To infer evidence of a causal effect approaches like the LCV model (O’Connor and Price 2018) can be applied. This approach estimates the co-kurtosis of the bivariate distribution of each trait’s marginal variant effects and assumes that a latent variable mediates the relationship between the two traits of interest. If the variant effects on trait one are proportionally larger than trait two, this can suggest partial genetic causality between the first and second traits. This posterior mean genetic causality proportion (GCP) is calculated from the normalised effect of the latent variable on the traits analysed and is reflective of the degree of the causal effect of one trait upon the other. Negative GCP values, provided they are of sufficient magnitude as indicated by|GCP| >0.6, suggest that trait two may causally influence trait one, while positive GCP values imply trait one may causally influence trait two. MR (Davies et al. 2018) uses a subset of independent genome-wide significant variants as assumed to be randomly distributed instrumental variables (IVs) to predict the effect of one trait on another. If these IVs are valid, they will index the effect of the exposure variable on the outcome of interest. Multiple MR methods exist, which make different assumptions about the behaviour of variants as valid IVs. Their results can be triangulated to make overarching inferences about the nature of the causal relationship between traits. Although a well-powered AN GWAS was published three years ago, there are limited articles which use these techniques for AN. At the time of writing, many papers have applied LDSC while significantly fewer have applied LCV and MR.

### Genetic correlation

LDSC is used to estimate genetic correlation between traits and thus makes no assumptions or adjustments for the presence of confounding which may be caused by factors such as LD or horizontal pleiotropic effects. The genetic correlation between a wide range of traits and AN has been investigated using LDSC. One study alone tested this association for 447 traits, identifying 46 associations including fasting insulin (FI) which was significantly negatively correlated with AN (Watson et al. 2019). Another measured the association between 50 biochemical traits and AN, 17 of these traits passed Bonferroni significance (*P* < 0.001) and were negatively correlated with AN, including C reactive protein (CRP) and glucose level (Reay et al. 2022). Two LDSC analyses have been performed for substance use, one compared to AN (Jang et al. 2022), and one for eating disorders in general (Munn-Chernoff et al. 2021). The eating disorder LDSC identified positive correlations with cannabis initiation and negative correlations with alcohol dependence however this was not replicated in the AN analysis. Determining which traits are associated with AN may provide information about shared biological mechanisms, which could inform future investigations of disease pathology.

#### Directional dependency

Directional dependency is a method to assess the likely causal direction between two traits (Pornprasertmanit and Little 2012). This approach utilises the kurtosis between variables and skewness to infer if a causal relationship between the traits is instigated from one variable or the other. The LCV approach applies the directional dependency principles to estimate the degree of partial genetic causality between two traits and which trait is the likely exposure (O’Connor and Price 2018). LCV has previously investigated the relationship between AN, substance use (Jang et al. 2022), CRP (Reay et al. 2022) and FI level (Adams et al. 2021). While only CRP displayed a significant GCP with AN, suggesting a negative association between circulating CRP and AN liability, this does not discredit a relationship existing between the other traits. The ability of LCV to infer evidence of a causal relationship between traits is limited by the model assumptions. The model assumes one intermediatory variable mediates the relationship between the two traits and may be biased if additional intermediatory variables are present. This approach cannot integrate the effects of known confounders into the model to estimate conditional effects. False positives from directional dependency methods will increase if uncorrelated traits are analysed. Finally, the LCV GCP does not estimate the magnitude of associations between traits and thus subsequent methods should be applied.

#### Causal inference

**Fig. 2:**
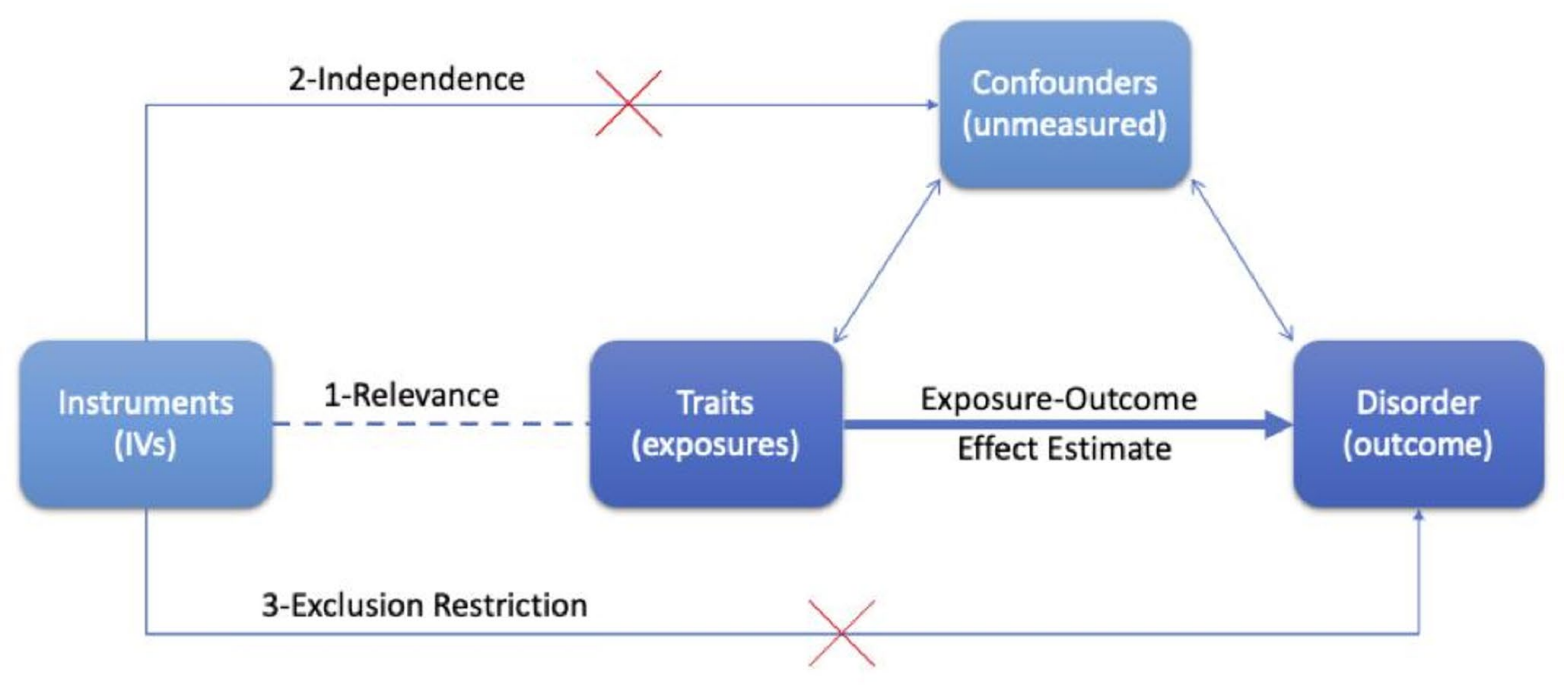
Mendelian randomisation directed acyclic graph. Three core assumptions of Mendelian randomisation are indicated: (1) Relevance, (2) Independence and (3) Exclusion restriction

MR is a statistical genetics method which utilises genetic variants as IVs to investigate evidence of a causal relationship between traits (Bowden et al. 2015, 2016). IVs are strongly associated with the risk factor of interest (exposure) and may be considered a proxy of lifetime exposure to this factor by modelling the association between variants associated with the risk factor and AN (outcome). IVs can be used to investigate causality by applying strict assumptions related to the association between IVs and other factors. The three core assumptions are illustrated in Fig. 2: (1) (Relevance) The IVs are correlated with the exposure. (2) (Independence) The IVs are not associated with any confounders that influence the relationship between the exposure and the outcome and (3) (Exclusion Restriction) The IVs do not directly influence the outcome. This 3rd assumption is what distinguishes IVs from proxy variables. All three of these assumptions must be satisfied for IVs to be considered valid for MR. Typically the 1st assumption is satisfied by choosing independent genome-wide significant variants for the exposure as IVs, however, the 2nd and 3rd assumptions can only be tested indirectly. Tests can check for effect estimate heterogeneity between variants and outliers can be removed, while for other methods concurrence of effect estimates across methods which hold different assumptions strengthens the evidence that these assumptions are not violated (de Leeuw et al. 2022). Thus, a well-conducted MR analysis that triangulates results across methods has the potential to accurately estimate a causal relationship between traits before an intervention trial is required. Variants are randomised at conception and thus the distribution of these risk-associated variants should be random throughout the population. Because of the random distribution of variants and the use of IVs to proxy exposure effects, MR is sometimes described as emulating a RCT (Sanderson et al. 2022).

Compared to other psychiatric disorders, relatively few MR analyses have been performed for AN. 35 MR studies on AN have been published at the time of writing (Table 3), these were identified through the advanced search feature of pubmed ((mendelian randomisation) OR (mendelian randomization) AND (anorexia nervosa)). Of these, 9 studies did not identify any significant results, from the remaining 26 studies, 36 traits were reported to be significantly associated with AN. 24 of these associations were significant after correcting for the number of tests performed, and 13 out of the 36 results reported as significantly associated, did not perform sensitivity analysis, or their sensitivity analysis suggests the potential for invalid instruments within their MR models. Interestingly, a significant association from an LCV analysis suggests a high degree of genetic causality between CRP and AN and presented evidence of a potentially protective effect of CRP from MR (Table 3). However this finding (OR = 0.908, [95% CI = 0.83–0.99], *P* = 0.029) (Reay et al. 2022) does not pass multiple testing correction if adjusted for the three other psychiatric disorder outcomes investigated. Similarly, the conditional multivariable MR association of IL-6 on AN is only nominally significant (OR = 0.96, [95% CI = 0.93–0.99], *P* = 0.037). Novel causal relationships have been reported for metabolic syndrome and 25-hydroxyvitamin D3 (Baumeister et al. 2023; Gao et al. 2024; Ye et al. 2021). Metabolic syndrome has evidence of a potential risk increasing effect on AN, while this study also reported evidence of a bidirectional relationship, however, the specific mechanisms of this were not investigated further (Gao et al. 2024). 25-hydroxyvitamin D3 potentially exerts a protective effect on AN, which was observed with the 2017 AN GWAS (Ye et al. 2021) but this was not replicated with the larger sample size 2019 GWAS which may indicate a false positive result within the first analysis with the lower powered GWAS (Baumeister et al. 2023). Twelve of the studies reported in Table 3 needed to increase their p value threshold (from *P* < 5 × 10^− 8^) for IV inclusion so that IVs that were not genome-wide significant could be included, which may indicate that the GWAS data is of insufficient strength for the first MR assumption and so should be treated cautiously. Without careful consideration of the assumptions of MR tests, the MR method provides insufficient evidence to estimate a causal relationship between two variables (Skrivankova et al. 2021).

Seven associations in particular have the strongest evidence of a putative unidirectional causal relationship between an exposure and AN (Table 3): worry (Lloyd et al. 2020), educational attainment (Chen et al. 2024; Yuan et al. 2021), FI (Adams et al. 2021), superior longitudinal fasciculus axial diffusivity (Song et al. 2021), attention deficient hyperactive disorder (Meisinger and Freuer 2023) and sphingomyelins (Jia et al. 2023). Note the two studies for educational attainment utilised different GWAS for the exposure. These risk factors may be involved in AN liability, while further analysis is required to explore the potential mechanisms of action. The predicted direction of effect is an important statistic when considering the potential implications on disease risk. For the case of educational attainment (Table 3), increased educational attainment is predicted to increase the risk of AN, however, decreased educational attainment may not be a desired intervention. FI is a pharmacologically modifiable trait with evidence, previously reported by the author, of a causal relationship (protective) with AN from MR where sensitivity analyses do not invalidate the assumptions of MR. Interestingly, there have been no RCTs that apply FI as a treatment to AN although one has applied recombinant human insulin-like growth factor-1 in conjunction with transdermal 17-beta estradiol to improve bone mineral density in adolescent AN patients (Singhal et al. 2021). This did not show an improvement, for the primary outcome of bone mineral density (AN status did not change), above the baseline estradiol treatment, suggesting insulin-like growth factor-1 does not improve AN symptoms.

Increased sphingomyelin levels are associated with increased AN risk from MR (Jia et al. 2023). Various drugs have been shown to target sphingomyelins such as; chemotherapy agents (Kroll et al. 2020), antidepressants and scyphostatin (Bi et al. 2021; Canals et al. 2011). Some of these are not ideal drug targets, such as daunorubicin (Shaw et al. 2018) which produces severe side effects. Drugs such as antidepressants; desipramine and fluoxetine, have shown success in reducing purging behaviour and depressive symptoms in bulimia nervosa and induce less severe side effects (1992; Yu et al. 2023). Sphingomyelins break down into sphingolipids which form structures within cellular membranes, the dysregulation of which is associated with cancers and immunity (Lee et al. 2023). One study proposed that sphingomyelin degradation in AN patients leads to hypercholesterolemia and increased cancer risk (Gizzi et al. 2022). While another found that intracerebroventricular injections of sphingomyelins in birds decreased food intake (Emoto et al. 2006). Additionally, studies show a relationship between immune dysregulation and immune disorders and AN, however further research is required to distinguish these effects from those observed due to malnutrition (Gibson and Mehler 2019). These results indicate that targeting sphingomyelins may have potential for the treatment of AN. Due to the variety of traits associated with sphingomyelin dysregulation, care should be taken to avoid unintended effects from targeting this cellular component. There appear to be no other RCTs that directly implement any of the traits with significant results investigated with MR, however, not all of these are suitable for intervention. An important function of MR is to provide evidence supporting RCTs, thus it is vital that when statistical evidence of a causal relationship accumulates, it is investigated through RCTs.

**Table 3:** Summary of anorexia nervosa Mendelian randomisation (MR) results to date (February 2024). 35 MR analysis for anorexia nervosa

Trait	OR	95% CI	*P*	Multiple testing correction	IV validity	Comments			Non-significant traits used as exposures for AN	Year
Body mass index	0.96	0.93–0.99	8.7 × 10^− 3^	Yes	No	HEIDI was used for outlier removal. No other sensitivity analysis was applied.			Type 2 diabetes	2019 (Watson et al. 2019) #
Body mass index*	0.76	0.66–0.86	3.8 × 10^− 3^	Yes	No					
--	NA	NA	NA	NA	NA	Heterogeneity in dependent instruments outlier method was used to remove outliers. No other sensitivity analysis was applied.			Body fat mass Body fat percentage	2019 (Hübel et al. 2019) #
--	NA	NA	NA	NA	NA	Only used a single IV. No additional methods were performed.			Caudate size Hippocampus size Putamen size Intracranial volume	2019 (Walton et al. 2019) #
Worry	1.79	1.25–2.55	1.0 × 10^− 3^	Yes	Yes	Extensive sensitivity analysis was performed. **Sensitivity analysis did not suggest evidence of any assumption violations.**			Anxiety disorders	2020 (Lloyd et al. 2020)
Fasting insulin adjusted for body mass index	0.48	0.33–0.71	2.27 × 10^− 4^	Yes	Yes	Extensive sensitivity analysis was performed. **Sensitivity analysis did not suggest evidence of any assumption violations.**			Glucose Glycated haemoglobin	2021 (Adams et al. 2021)
Educational attainment	1.72	1.5–1.97	1.16 × 10^− 14^	Yes	Yes	Multiple sensitivity analysis was performed. Multivariate MR was used to adjust for confounders. **Sensitivity analysis did not suggest evidence of any assumption violations.**			-	2021 (Yuan et al. 2021)
--	NA	NA	NA	NA	NA	HEIDI was used for outlier removal. No other sensitivity analysis was applied.			Birthplace Current population density	2021 (Maxwell et al. 2021) #
--	NA	NA	NA	NA	NA	Multiple sensitivity analysis was applied			Urate	2021 (Zhao et al. 2021)
--	NA	NA	NA	NA	NA	Extensive sensitivity analysis was performed.			Smoking initiation Smoking heaviness	2021 (Lloyd et al. 2021)
Superior longitudinal fasciculus axial diffusivity	0.62	0.5–0.76	6.4 × 10^− 6^	Yes	Yes	Extensive sensitivity analysis was performed. **Sensitivity analysis did not suggest evidence of any assumption violations.**			110 diffusion tensor imaging measurement and brain region volumes	2021 (Song et al. 2021)
Neuroticism	1.39	1.19–1.62	3.89 × 10^–5^	Yes	No	IVs used *P* < 1 × 10^− 5^. HEIDI was used for outlier removal. No other sensitivity analysis was applied.			-	2021 (Zhang et al. 2021) #
Leptin	0.39	0.24–0.64	1.5 × 10^− 4^	Yes	No	Used F > 10 rather than *P* < 1 × 10^− 8^ to select IVs (*N* = 4). Extensive sensitivity analysis was performed. MR Egger estimate direction of effect differs to the IVW method			-	2021 (Peters et al. 2021)
25-hydroxyvitamin D3	0.98	0.960–0.996	0.036	Yes	Yes	Used 2017 AN GWAS. Some sensitivity analysis performed. **Sensitivity analysis did not suggest evidence of any assumption violations.**			-	2021 (Ye et al. 2021)
TXNDC12	1.12	1.08–1.16	2.35 × 10^− 10^	Yes	Yes	IVs used *P* < 1 × 10^− 5^ Multiple sensitivity analysis was performed. Multivariate MR was used for confounders. **Sensitivity analysis did not suggest evidence of any assumption violations.**			2994 plasma proteins	2022 (Yang et al. 2022)
ADH1B	0.89	0.85–0.93	2.99 × 10^− 7^	Yes	Yes					
Eotaxin	1.32	1.049–1.675	0.018	No	Yes	--		Multiple sensitivity analysis was performed. **Sensitivity analysis did not suggest evidence of any assumption violations.**	37 cytokines	2022 (Chen et al. 2022)
TNFa	0.77	0.626–0.97	0.026	No	Yes	IVs used *P* < 1 × 10^− 5^				
IL-8	1.27	1.053–1.542	0.013	No	Yes					
FGFBasic	0.40	0.261–0.622	4.0 × 10^− 5^	Yes	Yes					
C reactive protein	0.90	0.83–0.99	0.029	No	Yes	Multiple sensitivity analysis was performed. Multivariate MR was used for confounders; BMI and IL6, IL6R. MVMR nominally significant for CRP conditional on IL6 and IL6R in some models. **Sensitivity analysis did not suggest evidence of any assumption violations**			-	2022 (Reay et al. 2022)
Ever smoked*	0.97	0.93–1.01	0.0374	No	Yes	Multiple sensitivity analysis was performed. **Sensitivity analysis did not suggest evidence of any assumption violations.**			Smoking habits Drinking habits	2022 (Jang et al. 2022)
--	NA	NA	NA	NA	NA	IVs used *P* < 5 × 10^− 6^ Multiple sensitivity analysis was performed.			ER + and ER- breast cancer*	2023 (Ren et al. 2023)
Ankylosing spondylitis	1.32	1.07–1.64	9.43 × 10^− 3^	Yes	No	Excluded SNPs associated with the outcome (*P* < 0.05). *The removal of these SNPs may induce bias into the MR model.* Multiple sensitivity analysis performed. **Sensitivity analysis did not suggest evidence of any assumption violations.**			-	2023 (Zuo and Li 2023)
Sphingomyelins	1.12	1.06–1.19	2.34 × 10^− 5^	Yes	Yes	Some sensitivity analyses were performed. **Sensitivity analysis did not suggest evidence of any assumption violations.**			91 blood metabolites	2023 (Jia et al. 2023)
Class Actinobacteria ID:419	1.53	1.19–1.96	8.9 × 10^− 4^	No	Yes	IVs used *P* < 1 × 10^− 5^. Multiple sensitivity analysis performed. **Sensitivity analysis did not suggest evidence of any assumption violations.**			210 gut microbiotas	2023 (Xia et al. 2023)
Attention deficient hyperactive disorder	1.28	1.11–1.47	0.001	Yes	Yes	Some sensitivity analyses were performed. **Sensitivity analysis did not suggest invalid instruments.**			-	2023 (Meisinger and Freuer 2023)
Blood selenium	0.87	0.77–0.97	0.016	Yes	No	Used 2017 AN GWAS. Performed multiple sensitivity analysis. Evidence of potentially invalid instruments from sensitivity analysis.			Blood-toenail selenium levels	2023 (Guo et al. 2023)
--	NA	NA	NA	NA	NA	Multiple sensitivity analyses were performed.			25- hydroxyvitamin D3	2023 (Baumeister et al. 2023)
TIMP4	0.83	0.76–0.91	9.7 × 10^− 5^	No	Yes	3 IVs available across three studies. Minimal sensitivity analysis performed. **Sensitivity analyses do not suggest invalid instruments.**			1610 circulating proteins	2023 (Lu et al. 2023)
--	NA	NA	NA	NA	NA	Less than 4 IVs for each exposure. Minimal sensitivity analyses were performed.			6 types of polyunsaturated fatty acids	2023 (Nomura et al. 2023)
PDE9A*	0.93	0.870–0.993	0.032	No	No	IVs used *P* < 1 × 10^− 5^. Some sensitivity analysis performed. The MR weighted mode method suggests potential assumption violation.			8 Phosphodiesterases	2023 (Jiang et al. 2023)
Plasma caffeine	1.12	1.024–1.238	0.039 (FDR)	Yes	No	IVs used *P* < 5 × 10^− 5^. Located near *CYP1A2* and *AHR* rather than genome-wide. Minimal sensitivity analysis. Sensitivity analysis suggest there may be invalid instruments			-	2023 (Woolf et al. 2023)
Type 2 diabetes	-	-	0.049	No	No	Unclear how their IVs were selected (e.g. number of IVs or p value threshold). State that they adjusted for pleiotropy but provide no explanation of how and do not perform any additional sensitivity analysis. Do not report their effect estimates.			-	2023 (Ding et al. 2023) #
Type 2 diabetes *	-	-	0.0063	No	No					
Body mass index	1.43	1.3–1.58	5.71 × 10^− 13^	Yes	No	**Sensitivity analysis suggest no assumption violations**	Removed SNPs associated with confounding variables or the outcome (*P* < 5 × 10^− 8^). *The removal of these SNPs may induce bias into the MR model.* Incorrectly assigned effect and other allele to anorexia nervosa (used REF as the effect allele) which results in effect estimates for anorexia nervosa in the reverse direction to expected. **Performed minimal sensitivity analysis which do not suggest evidence of invalid instruments**		Waist to height ratio adjusted for body mass index	2023 (Chen et al. 2023)
Waist to height ratio	1.29	1.15–1.45	1.21 × 10^− 5^	Yes	No	Sensitivity analysis suggests the presence of invalid instruments				
Atopic dermatitis*	1.1	1.068–1.134	4.45 × 10^− 10^	Yes	No	IVs used *P* < 1 × 10^− 5^ Excluded SNPs associated with confounders or SNPs associated with the outcome (*P* < 5 × 10^− 8^). Removed SNPs with F < 50. *The removal of these SNPs may induce bias into the MR model.* Otherwise performed multiple sensitivity analysis **Sensitivity analyses do not suggest invalid instruments.**			Atopic dermatitis	2024 (Cao et al. 2024)
--	NA	NA	NA	NA	NA	IVs used *P* < 5 × 10^− 6^ Performed some sensitivity analyses.			Focal epilepsy*	2024 (Chu et al. 2024)
Chronotype*	1.04	1.005–1.074	0.02	No	Yes	Only 8 IVs with AN as the exposure. Some sensitivity analysis performed. **Sensitivity analysis did not suggest invalid instruments.**			Daytime napping, Daytime sleepiness, Insomnia, and Sleep duration	2024 (Wilcox et al. 2024)
Educational attainment	1.47	1.22–1.76	4.19 × 10^− 9^	Yes	Yes	-	Multiple sensitivity analysis was performed. **Sensitivity analysis did not suggest evidence of any assumption violations.**		45 lifestyle factors 2992 plasma proteins	2024 (Chen et al. 2024)
STOM	0.9	0.85–0.95	7.52 × 10^− 5^	No	Yes	IVs used *P* < 1 × 10^− 5^				
ADH1B	0.89	0.85–0.94	4.13 × 10^− 6^	Yes	Yes					
Metabolic syndrome	1.42	1.25–1.61	1.9 × 10^− 7^	Yes	Yes	-	Multiple sensitivity analysis was performed. **Sensitivity analysis did not suggest evidence of any assumption violations.**		-	2024 (Gao et al. 2024)
Metabolic syndrome*	1.01	1.01–1.02	8.72 × 10^− 4^	Yes	Yes	IVs used *P* < 5 × 10^− 5^				

Columns indicate statistics for any significant traits (trait, odds ratio (OR), 95% confidence interval (CI): Lower bound - Upper bound, p value (P)), any non-significant traits and the year of publication. Multiple testing correction indicates ‘Yes’, ‘No’ or ‘NA’ based on if the reported significant association passed Bonferroni multiple testing correction. IV validity indicates ‘Yes’, ‘No’ or ‘NA’ based on if the sensitivity analysis of reported significant results provided supporting evidence that valid IVs were used. NA indicates that a significant result was not reported. Comments indicate any notes related to the analysis and the extent of sensitivity analysis performed. Bold text represent analysis which have no evidence of MR assumption violation.

* indicates that reverse causality direction of effect is investigated (anorexia nervosa influencing the exposure),

# indicates a method besides the inverse variance weighted method was used as the primary reported analysis method.

### Limitations for the use of genetic data to study anorexia nervosa

While the identification of potential AN risk factors reveal information about potential mechanisms of disease action, there is more that can be done to uncover the functional relevance of disease-associated processes. Large cohorts are required to detect smaller effects and rare variant effects, however, AN patients often have high rates of treatment non-compliance and are thus hard to recruit (Túry et al. 2019). Additionally, males with AN present differently from females with the disorder and are underdiagnosed compared to their female counterparts (Strother et al. 2012). More male AN patients need to be recruited so that better powered male specific AN GWAS can be performed and determine variants associated with sex-specific risk factors (Culbert et al. 2021). A large issue prevalent through many GWAS analyses is the lack of non-European cohorts (Peterson et al. 2019). Currently, the only GWAS-based analyses of AN, such as MR, are from European individuals and thus any conclusions reached cannot necessarily be extended to individuals of non-European ancestry. More causal risk estimation analyses, such as MR and LCV, should be performed for pharmacologically actionable exposures relevant to disease pathology to help expedite the discovery of pharmacological treatments for AN. One limitation of this is that it requires the availability of large GWAS for these pharmacologically actionable exposures. Additionally, the availability of larger GWAS for risk factors and AN will improve the estimation of variant effect associations and as a corollary, gene-based and trait-based association estimates. Critically, when genetic analyses provide evidence to support a causal relationship between potential treatments, intervention studies need to be conducted to utilise these results, or else progress to improve patient outcomes will not occur.

## Conclusions

Recently published AN GWAS provide a promising opportunity to investigate the pathophysiology of this disorder but has yet to be utilised to its full potential. There are many existing and emerging analytical methods which could be applied to the available AN genetic data. These may be able to explain a component of the heritability of AN leading to the discovery of new treatment opportunities, which have been missed through conventional analyses. The accurate prioritisation of causal variants related to disease risk and correctly identifying the pathways through which these variants influence trait risk is vital to progressing understanding of disease pathology. The genetic correlation between AN and other related phenotypes can provide insight into associated mechanisms related to trait risk while techniques which dissect the causal relationship between traits may reveal potential risk factors which may be modulated to influence disease risk. More research is required to improve the application of each of these methods and investigate the biological implications of their results to reveal unique aspects of the aetiology and pathophysiology of this disease.

## Data Availability

No datasets were generated or analysed during the current study.
